# Design and Development of a Polymeric-Based Curcumin Nanoparticle for Drug Delivery Enhancement and Potential Incorporation into Nerve Conduits

**DOI:** 10.3390/molecules29102281

**Published:** 2024-05-12

**Authors:** Giuliana Gan Giannelli, Edwin Davidson, Jorge Pereira, Swadeshmukul Santra

**Affiliations:** 1 NanoScience Technology Center, University of Central Florida, Orlando, FL 32826, USA; giuliana.giannelli@ucf.edu (G.G.G.); edavidson@ucf.edu (E.D.); jorge.pereira@ucf.edu (J.P.); 2 Burnett School of Biomedical Sciences, University of Central Florida, Orlando, FL 32826, USA; 3 Department of Chemistry, University of Central Florida, Orlando, FL 32826, USA

**Keywords:** curcumin nanoparticles, nanodelivery, tannic acid, polyvinylpyrrolidone, nerve conduits, peripheral nerve regeneration, oxidative stress

## Abstract

Peripheral nerve injuries (PNI) impact millions of individuals in the United States, prompting thousands of nerve repair procedures annually. Nerve conduits (NC) are commonly utilized to treat nerve injuries under 3 cm but larger gaps still pose a challenge for successful peripheral nerve regeneration (PNR) and functional recovery. This is partly attributed to the absence of bioactive agents such as stem cells or growth factors in FDA-approved conduits due to safety, harvesting, and reproducibility concerns. Therefore, curcumin, a bioactive phytochemical, has emerged as a promising alternative bioactive agent due to its ability to enhance PNR and overcome said challenges. However, its hydrophobicity and rapid degradation in aqueous solutions are considerable limitations. In this work, a nanoscale delivery platform with tannic acid (TA) and polyvinylpyrrolidone (PVP) was developed to encapsulate curcumin for increased colloidal and chemical stability. The curcumin nanoparticles (CurNPs) demonstrate significantly improved stability in water, reduced degradation rates, and controlled release kinetics when compared to free curcumin. Further, cell studies show that the CurNP is biocompatible when introduced to neuronal cells (SH-SY5Y), rat Schwann cells (RSC-S16), and murine macrophages (J774 A.1) at 5 μM, 5 μM, and 10 μM of curcumin, respectively. As a result of these improved physicochemical properties, confocal fluorescence microscopy revealed superior delivery of curcumin into these cells when in the form of CurNPs compared to its free form. A hydrogen peroxide-based oxidative stress study also demonstrated the CurNP’s potential to protect J774 A.1 cells against excessive oxidative stress. Overall, this study provides evidence for the suitability of CurNPs to be used as a bioactive agent in NC applications.

## 1. Introduction

Currently, 2–3% of all patients admitted to level 1 trauma centers in the United States exhibit some form of peripheral nerve injury (PNI) [1]. As a result, approximately 200,000 nerve repairs are performed annually in this country alone [2]. While there is an abundance of literature on peripheral nerve regeneration (PNR), there is still a lack of effective treatments for achieving full functional recovery [3]. For perspective, a meta-analysis of 623 individuals who underwent microsurgical repair revealed that only 51.6% and 42.6% of patients achieved satisfactory motor and sensory recovery, respectively [4]. Life-long debilitation, therefore, afflicts the patient group with less satisfactory outcomes as they live with issues including neuropathic pain, numbness, or paralysis, to name a few.

Although nerves in the peripheral nervous system can regenerate, they do so at a slow rate of 1–2 mm a day [5]. Further, the process can be hindered in some severe cases of injury because of fibrosis, delayed axonal regrowth, chronic inflammation, and oxidative stress [6]. For treatment, the gold standard remains end-to-end nerve suturing, but this approach is suitable only for lesions that do not have a significant gap between the proximal and distal ends of the injured nerve. When a relatively large gap does exist, an autologous nerve graft is typically used to bridge the two ends [3]; while this was established as the ideal treatment option for such cases, issues such as sourcing a nerve graft from the patient’s own body and the potential for further injury, incompatible diameters of donor and injured nerves, and length limitations of the donor graft remain great challenges.

In the past two decades, nerve conduits (NC) have, therefore, emerged as an attractive alternative to autologous nerve grafts for treating PNI [7]. However, since their approval by the FDA and introduction to the market in 1999, the extent of their application has been limited to injured nerve segments of 3 cm and under [2]. As a result, some recent studies have suggested embedding bioactive agents into NCs as a viable strategy to improve their efficacy and further enhance PNR [8,9,10,11,12]. For instance, phytochemicals (e.g., curcumin, ascorbic acid, green tea polyphenols, quercetin, and ursolic acid) have demonstrated their potential to promote PNR following PNI in animal studies [5,13,14,15]. These compounds demonstrated notable improvements in functional recovery compared to mock conditions through assessments of nerve conduction velocity, nerve compound action potential, axon regeneration, axon remyelination, and sciatic function index. However, many of these studies utilized free-form phytochemicals, which are sub-optimal due to issues such as hydrophobicity and rapid degradation in solution. The hydrophobic nature of compounds reduces their bioavailability, while their short half-life renders them unsuitable for NCs as the application would require slow and sustained release of active ingredients over prolonged periods.

Curcumin is one of the most widely studied phytochemicals in the context of this application and was proven to be effective for treating PNI in in vivo studies [13,16,17,18,19]. It is a polyphenol with well-established antioxidant, anti-inflammatory, antimicrobial, anticancer, and neuroprotective properties [20,21,22,23,24]. However, as alluded to earlier, curcumin is hydrophobic and readily degrades in solution, thus reducing its long-term stability, bioavailability, and overall efficacy [25]. In order to overcome these challenges and optimize drug delivery, a nanoformulation comprising tannic acid (TA) and polyvinylpyrrolidone (PVP) was developed to encapsulate curcumin. TA (an antioxidant excipient) and PVP (a hydrophilic excipient) were selected to capture curcumin and improve curcumin’s colloidal stability in water, respectively [26,27]. As with many other nanoparticle (NP) systems, the CurNP forms through self-assembly, which in turn is driven by hydrogen bonding, pi-pi stacking, and hydrophobic interactions between the PVP, TA, and curcumin [28,29]. Further, PVP has already received FDA approval as an excipient while TA is generally recognized as safe, thus allowing for easier translation into NC applications [27,30]. It should be noted that despite the potential of curcumin NPs in treating PNI, only a single study has so far been identified that utilizes curcumin-encapsulated chitosan nanoparticles for this application [31].

Herein, we demonstrate the successful development of a polymeric nanoplatform for the encapsulation of curcumin. The results show that the nanoplatform offers curcumin protection against degradation, sustained release over time, and improved stability in aqueous solutions. The enhanced physicochemical properties of CurNPs compared to free curcumin led to a significant improvement in delivery into PNR cell models (SH-SY5Y, J774 A.1, and RSC-S16). Further, macrophages treated with the CurNPs exhibited increased protection against hydrogen peroxide-induced oxidative stress. These findings provide justification for CurNPs to be further developed and embedded into NCs for potential in vivo studies of PNI and repair.

## 2. Results and Discussion

### 2.1. Material Characterization

The CurNPs were synthesized through a flash precipitation method whereby an ethanolic curcumin–TA solution was added dropwise into an aqueous PVP solution under constant magnetic stirring. This optimized protocol resulted in a homogenously opaque yellowish-orange solution indicative of curcumin’s improved colloidal stability (Figure S1). It should be noted that the synthesis method underwent a series of iterations to render the highest encapsulation efficiency (EE) of curcumin. Initially, the molar ratios of the PVP and TA were increased relative to curcumin, resulting in a gradual increase in EE from 36% to 68%, at which point a plateau was observed, as summarized in Table S1. The plateau in EE was overcome by decreasing the overall synthesis volume from 15 mL to 10 mL and further increasing the molar ratio of TA-PVP to curcumin, as shown in Table 1. This led to an EE of 88%, creating the CurNP system that is referenced throughout this work. It is worth noting, however, that this EE is comparable to that in other recently reported work [32,33,34]. However, these studies achieved similar EE with a much lower concentration of captured curcumin, that is, in the range of 1 to 700 ppm [35,36,37,38]. Hence, an advantage of the present work is that the NP system is able to capture up to 1100 ppm of curcumin.

**Figure 1:**
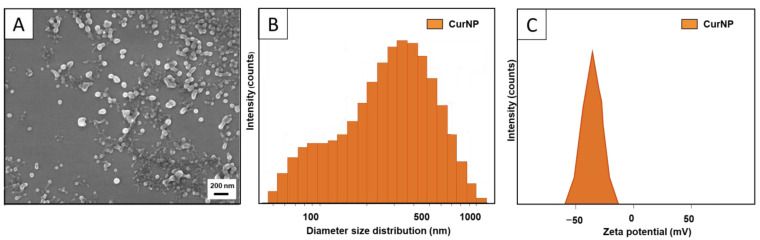
(**A**) SEM image demonstrates somewhat spherical CurNPs with some agglomeration. (**B**) DLS histogram demonstrates the hydrodynamic diameter (size) distribution of CurNPs, with notable polydispersity due to the agglomeration of primary particles. (**C**) Zeta potential charge distribution of CurNPs in an aqueous solution.

The SEM image (Figure 1A) demonstrates spherical NPs ranging from 40 to 120 nm, with some agglomeration likely due to the preparation process (centrifugation and drying) that samples are subjected to for image acquisition. Additionally, the dynamic light scattering (DLS) results revealed the average hydrodynamic diameter of the CurNPs to be 220 nm with a Polydispersity Index of 0.3 (Figure 1B). It should also be noted that the hydrodynamic diameter of the CurNPs exists as a bimodal distribution, which explains the polydispersity of the particles. Considering that the SEM micrographs showed primary particles under 200 nm, it is likely that the larger size distribution demonstrated by the DLS data in Figure 1B is due to particle agglomeration in the colloidal state. The zeta potential of the CurNPs was found to be −35 mV, which, in part, explains the enhanced water stability of the NPs when compared to free curcumin (Figure 1C). The DLS of unloaded TA–PVP showed an average size of 302 nm and a similar zeta potential of −35 mV (Figure S2). This provides evidence that the TA and PVP form the carrier of the NP system which captures the curcumin. The larger average size of the unloaded TA–PVP carrier compared to CurNPs could be due to the higher overall hydrophilicity of the former, leading to increased water absorption and swelling. The integration of curcumin within the CurNPs increases the hydrophobicity of the particles, thus reducing the swelling. Nonetheless, the average sizes of the unloaded and loaded NPs are comparable. Collectively, these results demonstrate the successful synthesis of water-stable spherical NPs with a high encapsulation of curcumin. The PDI, however, can likely be improved upon in future studies by providing increased control over the drop rate or magnetic stirring speed during synthesis.

**Figure 2:**
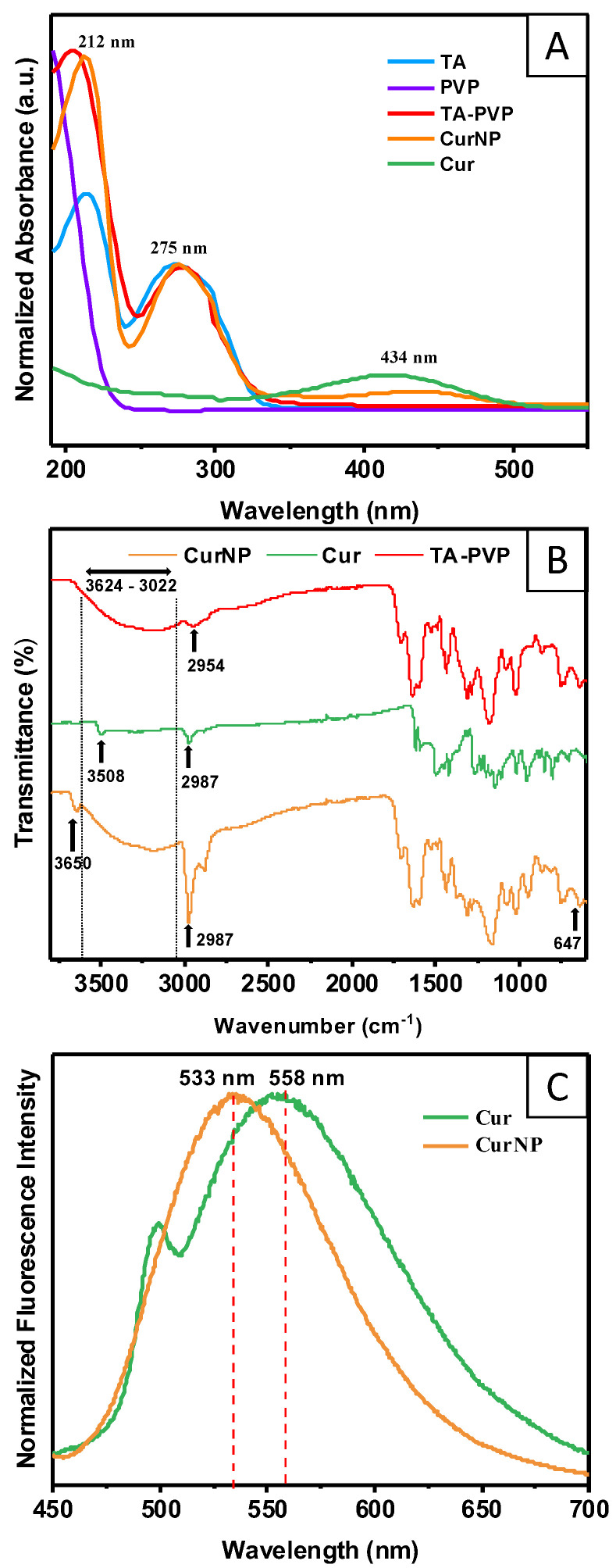
(**A**) UV–Visible spectra of curcumin, CurNP, and TA–PVP to confirm the presence of components within the NP system. (**B**) FTIR spectra of curcumin, CurNP, and TA–PVP to confirm the presence and interactions of components within the NP system. (**C**) Fluorescence emission spectra of CurNP and curcumin in an aqueous solution to determine localization of curcumin within the NP.

The UV–Visible spectra were recorded to discern the composition of the CurNPs and confirm the presence of curcumin, TA, and PVP after purification (Figure 2A). The CurNP spectrum exhibits a peak at 434 nm, which is characteristic of curcumin. Additionally, the peaks at 212 and 275 nm in the CurNP spectrum correspond to the excipients of the nanoparticle platform (TA–PVP). Fourier Transform Infrared (FTIR) spectroscopy was also employed to further confirm the presence and interactions of these molecules within the CurNP system (Figure 2B). First, the CurNP spectrum showed broad O-H stretching from 3022 cm^−1^ to 3624 cm^−1^, which is typical for TA due to the many hydroxyl groups present in the molecule; this can also be seen in the TA–PVP spectrum. There is also an N-C=O bending at 647 cm^−1^ evident in both NP spectra, which can be attributed to PVP. Additionally, the phenolic O-H stretching peak typical for curcumin shifts from 3508 cm^−1^ to 3650 cm^−1^ when in the CurNP, suggesting that it is captured by the NP system in its viable form. Finally, sp^3^ C-H stretching is present in all spectra shown at roughly 2987 cm^−1^, with especially pronounced bands in the CurNP spectrum, likely due to the compounding effect of this chemical bond which is found in each molecule comprising the NP system. More information on the identified peaks in the spectra and the corresponding chemical groups can be found in Table S2. In summary, the CurNP UV–Visible and FTIR spectra exhibited the signature peaks for curcumin, TA, and PVP, which ultimately corroborates the suggested composition of the NP system.

Since curcumin exhibits inherent fluorescence when excited at 425 nm, this property was leveraged to assess its localization in the CurNP, whether towards a more hydrophilic PVP surface or the hydrophobic core of the NP. According to the principle of solvatochromism, a shift in the emission peak of a molecule corresponds to a change in its microenvironment or solvent [39]. The results here show that there was a significant shift in the peak emission from 558 nm to 533 nm when comparing curcumin in its free form versus curcumin captured by TA–PVP, respectively (Figure 2C). This notable blue shift suggests that curcumin exists within the relatively hydrophobic core of the NP given that the free curcumin exists in an aqueous environment. This finding is important as a large part of the motivation for this research was to effectively encapsulate and shield curcumin within the core of a carrier to protect it from degradation, as discussed in the following section. Overall, the results from the spectroscopy studies showcase the successful synthesis of TA–PVP NPs with viable curcumin encapsulated within the core.

### 2.2. Degradation Rate, Controlled Release, and Antioxidant Potential of CurNPs

**Figure 3:**
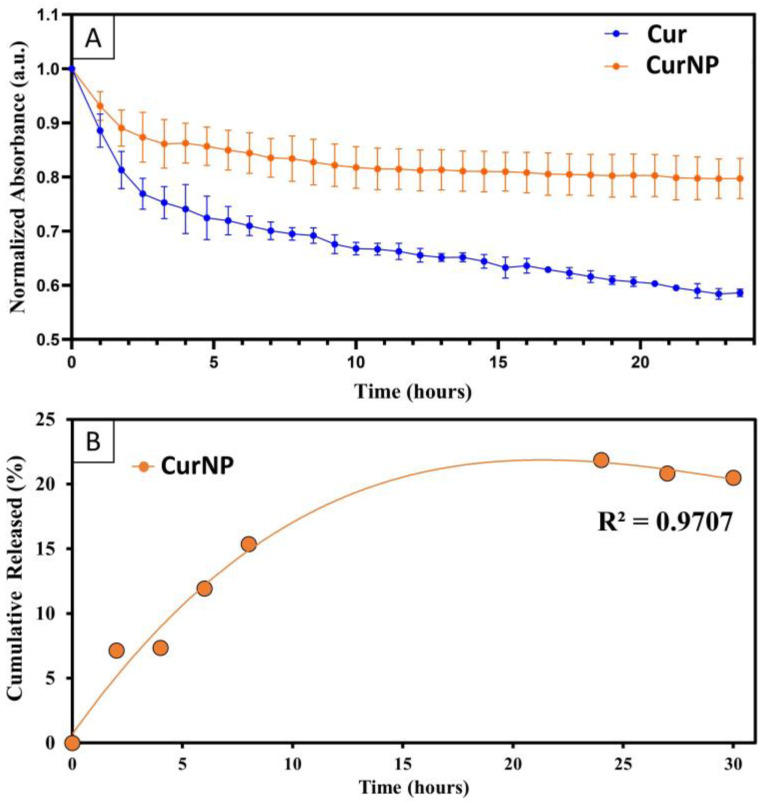
(**A**) Degradation rate measured through the absorbance of curcumin and CurNPs in PBS at 425 nm for 24 h. Statistical analysis was performed using simple liner regression (*p* < 0.0001). (**B**) Cumulative release of curcumin from CurNPs dispersed in water, measured up to 30 h.

As discussed earlier, one major limitation of curcumin as a therapeutic is its premature degradation due to oxidation or hydrolysis. Given the fluorescence emission spectra pointing to the successful capture of curcumin within the core of CurNPs, it was anticipated that the stability of the entrapped molecules would be greatly enhanced compared to those in free form. In this study, the degradation rate was assessed through spectrophotometric kinetic experiments by dispersing either free curcumin or CurNPs in PBS and measuring the absorbance every hour for 24 h. By the end of the 24 h, it was observed that only about 20% of curcumin had degraded when in the CurNP form, versus the 40% that had degraded when in free form (Figure 3A). Linear regression analysis showed a statistically significant difference between the slopes of the degradation curves (−0.01123 and −0.005019, for free curcumin and CurNPs, respectively). This emphasizes the NP system’s capacity to preserve curcumin in its viable form, which is ultimately important for enhancing its therapeutic potential.

The release rate of curcumin from the NP system was also assessed to determine the potential for slow and sustained release. CurNPs were dispersed in water and measured spectrophotometrically until a notable plateau was observed (Figure 3B). A maximum cumulative release of 20% of curcumin from the NPs was observed by the end of 20 h, comparable to the 20% of degraded curcumin observed in the degradation rate study. Given the similarity in these findings and the assumption that the curcumin released from the NPs is subsequently degraded, these experiments successfully corroborate one another. More importantly however, this experiment serves as a direct measure to demonstrate the ability for CurNPs to provide sustained release of curcumin over time [40,41]. It should be noted that there seems to be a decrease in the cumulative release of curcumin once a plateau is reached (after 24 h), and this is likely due to the degradation of the curcumin that is released by the NP system.

Considering how chronic oxidative stress following PNI can hinder successful PNR, it is imperative that a nanoformulation for the goal of NC incorporation also has intrinsic antioxidant properties to mitigate the excessive production of Reactive Oxygen Species (ROS) and Reactive Nitrogen Species (RNS). [16,38] For this reason, a 2,2-diphenyl-1-picryhyldrazyl (DPPH) radical scavenging activity assay was performed to determine the antioxidant capacity of the CurNP system. It was found that CurNPs and TA–PVP had a similar scavenging activity of 93% with respect to the negative control (Figure S3). They also demonstrated similar scavenging activity to ascorbic acid, a well-known antioxidant compound. In contrast, free curcumin had a significantly lower scavenging activity of 55%.

Overall, the evidence for protection against degradation, sustained release, as well as high antioxidant potential highlight the superiority of this NP system over curcumin in its free form.

### 2.3. In Vitro Viability Studies on Cell Models for PNI

This study was conducted to determine the in vitro safety profile of CurNPs with three different cell line models used to represent cells involved in PNI and subsequent PNR: S16 Rat Schwann Cells (RSC-S16), murine macrophages (J774A.1), and neuronal cells (SH-SY5Y). Schwann cells are instrumental during PNR as they provide physical (bands of Bungner) and chemical (growth factors) cues to the regenerating axons. Additionally, Schwann cells also signal for the influx of macrophages to the site of injury and together, they facilitate Wallerian degeneration, which is an important precursor to PNR [42,43,44]. SH-SY5Y cells were selected in this study as a substitute for primary neurons as they are commonly used as a cell model to study nerve regeneration [45,46].

**Figure 4:**
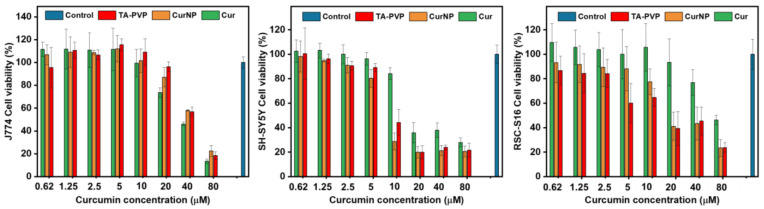
Viability of J774A.1, SH-SY5Y, RSC-S16 cells upon treatment with curcumin, CurNPs, and TA–PVP for 24 h, followed by AlamarBlue reagent for 2 h. Fluorescence was measured at Ex/Em 560/590 nm. Error bars correspond to a standard deviation (*n* = 9).

The viability of these cell lines when treated with CurNPs was measured using an alamarBlue assay. In this assay, metabolically active cells convert resazurin to resorufin whereby the latter can be detected via fluorescence at 590 nm [47]. In the case of TA–PVP-only conditions, the concentration range for these components was identical to the TA–PVP concentrations applied for CurNPs. Figure 4 demonstrates that the viability of SH-SY5Y cells remains relatively uncompromised when treated with TA–PVP or CurNP up to concentrations corresponding to 5 μM of curcumin, beyond which cytotoxicity becomes evident. Further, cell viability is significantly reduced by CurNPs at 10 μM, likely because both curcumin and TA were reported to be anti-cancer agents, and SH-SY5Y is a neuroblastoma cell line; this points to the importance of careful selection in cell lines to ensure compatibility with the therapeutic being tested. Future studies could consider the use of other immortalized neuronal cell lines such as 50B11, or even primary cells such as Dorsal Root Ganglions, as models instead [48]. Figure 4 also demonstrates that the CurNPs are biocompatible on J774A.1 and RSC-S16 cells up to concentrations corresponding to 10 μM and 5 μM of curcumin, respectively. These results provide evidence for the biocompatibility of the CurNP system on relevant cell lines. Additionally, they provided a reference for treatment concentrations to assess the in vitro delivery of curcumin into each cell type via confocal fluorescence microscopy.

### 2.4. In Vitro Cellular Delivery of Curcumin

**Figure 5:**
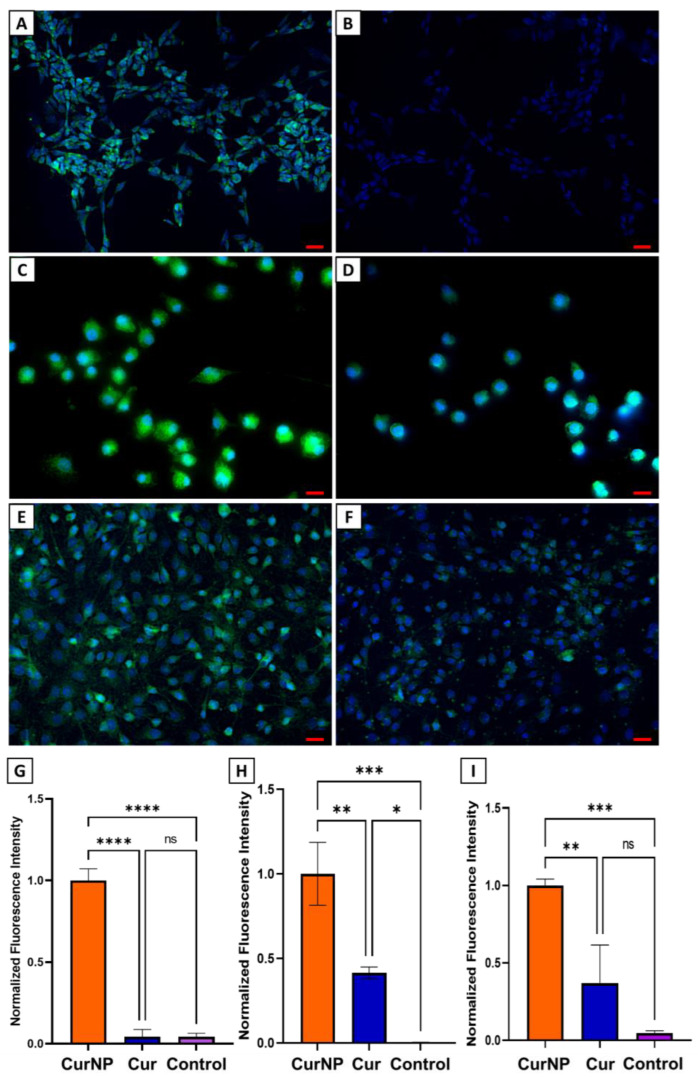
Confocal fluorescence microscopy images of (**A**) SH-SY5Y, (**C**) J774A.1, and (**E**) RSC-S16 upon treatment with CurNPs for 24 h (green channel). Images (**B**) SH-SY5Y, (**D**) J774A.1, and (**F**) RSC-S16 correspond to cells treated with free curcumin for 24 h. (**G**–**I**) Quantitative analysis of green fluorescence intensity in for SH-SY5Y, J774A.1, RSC-S16 cells, respectively (normalized). The red scale bar represents 20 µm. The bar graphs represent the mean and error bars correspond to the standard deviation (*n* = 3). Statistical analysis was performed using one-way ANOVA (* *p* < 0.05, ** *p* < 0.01, *** *p* < 0.001, **** *p* < 0.0001, ns not significant).

Confocal fluorescence microscopy studies were performed on SH-SY5Y, J774A.1, and RSC-S16 cells to assess the delivery of curcumin when administered in its encapsulated or free form. As mentioned, this was possible via the inherent fluorescence that curcumin exhibits, observable in the green channel. Figure 5 clearly demonstrates significantly higher green fluorescence intensity when cells are treated with CurNPs compared to free curcumin, after 24 h. It can be inferred that this is the direct result of curcumin’s increased colloidal stability and extended lifetime when encapsulated by TA and PVP. Again, this was shown by the degradation rate study whereby 20% more curcumin was retained in its viable form when encapsulated by the NP system over a period of 24 h. This study therefore provides direct evidence that the improved physicochemical attributes of curcumin offered by the NP platform leads to enhanced delivery into cells and provides justification for further investigation to assess potential improvements in therapeutic efficacy.

### 2.5. Hydrogen Peroxide (H_2_O_2_)-Induced Oxidative Stress and Cell Viability

For this study, the J774A.1 cell line was selected as a model due to the primary role of macrophages in inducing a pro-inflammatory microenvironment (M1 phenotype) by releasing ROS and RNS during the early stages of Wallerian degeneration [44]. While undesirable in the long term, this property is nonetheless important immediately following PNI to promote tissue necrosis and allow subsequent PNR. Later stages of Wallerian degeneration necessitate macrophages to then adopt an anti-inflammatory phenotype (M2) to facilitate PNR. Therefore, the ability of CurNPs to preserve macrophages (J774A.1) under excessive oxidative stress and ensure their longevity would be an important feature for NC applications.

J774A.1 cells were first pre-treated with CurNPs at concentrations up to 40 μM of curcumin, followed by the addition of 100 µM of hydrogen peroxide, a well-known ROS precursor. The results show that at a concentration corresponding to 10 µM of curcumin, there was a statically significant improvement in cell viability when cells were pre-treated with CurNPs, compared to the hydrogen peroxide-only control (Figure S4). It can also be observed that while the DPPH radical scavenging activity results demonstrate comparable antioxidant potential of TA–PVP and CurNPs, the results here show the superiority of the CurNPs in protecting cells compared to TA–PVP. This discrepancy can be explained by the fact that in the DPPH assay, measurements were made after only one hour, at which point most of the curcumin has yet to be released from the NP. In contrast, cell viability in this experiment was measured 24 h after the introduction of treatments, and it can be speculated that considerably more curcumin had been released compared to that in the DPPH assay. Further, it can be asserted that conditions in the DPPH assay led to a quick saturation in scavenging activity. Differences between TA–PVP and CurNPs in the context of a DPPH assay may therefore be better discerned by adding less treatments in future studies. Nevertheless, these results provide some evidence of effective protection against oxidative stress in a macrophage cell line. Altogether, these results showcase the promising potential of CurNPs as a suitable bioactive for NC incorporation to treat PNI.

## 3. Materials and Methods

### 3.1. Reagents

Curcuminoid powder (HPLC > 95%) was purchased from Alfa Aesar (Haverhill, MA, USA). Tannic acid (MW 1701), polyvinylpyrrolidone (MW 8000), L-ascorbic acid, 99.5% glycerol, Dulbecco’s Modified Eagle Medium (DMEM), DMEM/Nutrient Mixture-F12, penicillin–streptomycin (10,000 U/mL), fetal bovine serum (FBS), 0.5% EDTA-trypsin, phosphate-buffered saline (PBS), Alamar Blue, 4% paraformaldehyde and Hoechst were purchased from Thermo-Fisher Scientific (Waltham, MA, USA). SH-SY5Y (CRL-2266), S16 Schwann cells (CRL-2941), and murine macrophages J774A.1 (TIB-67) were purchased from ATCC (Manassas, VA, USA).

### 3.2. Synthesis of Curcumin Nanoparticles

The synthesis of CurNPs was performed with a flash precipitation method [28,49]. Firstly, 11 mg of curcumin powder was dissolved in 1 mL ethanol (95%) and sonicated. Then, 170 mg of TA was added to the curcumin–ethanolic solution and sonicated until all the powder had dissolved. Next, the curcumin–TA solution was added dropwise into 9 mL of aqueous PVP solution (9.4 mg/mL) under constant magnetic stirring at 1000 rpm. The mixture was left under magnetic stirring for an hour. The resulting molar concentrations of curcumin, TA, and PVP were 0.03 mM, 0.1 mM, and 0.001 mM, respectively.

### 3.3. Dynamic Light Scattering (DLS) and Zeta Potential (ZP)

Prior to DLS and ZP measurements, samples were diluted to approximately 200 ppm of curcumin and then analyzed using a Malvern Zetasizer ZS90 (Malvern Panalytical, Malvern, United Kingdom) to determine the hydrodynamic size distribution and surface charge.

### 3.4. Scanning Electron Microscopy (SEM)

The CurNPs and TA–PVP carrier were each imaged to evaluate their morphology. First, NPs we centrifuged at 10,000 rpm (Eppendorf 5810R [Fisher Scientific, Waltham, MA, USA]) for 10 min and resuspended DI water. This wash step was repeated twice. The solution was then diluted to approximately 200 ppm of curcumin, drop-casted onto a silicon wafer, and dried in a desiccator for 24 h. The samples were imaged under high vacuum using a Zeiss Nvision 40 (Zeiss, Oberkochen, Germany) with a 5 kV acceleration voltage.

### 3.5. UV–Visible (UV–Vis) Spectroscopy

The UV–Vis spectra of the samples were recorded using a Cary 300 Bio UV–Vis spectrophotometer (Agilent Technologies, Santa Clara, CA, USA) with 1 cm quartz cuvette. The samples were washed and diluted following the same protocol as noted for the SEM preparations.

### 3.6. Fourier Transform Infrared Spectroscopy (FTIR)

Prior to FTIR analysis, samples were washed once following the same protocol as noted for the SEM preparations and frozen at −20 °C overnight. The frozen samples were then lyophilized using a Labconco lyophilizer (Labconco, Kansas City, MO, USA) to obtain a dry powder for further analysis. The FTIR spectra of samples were recorded using a Perkin Elmer Spectrum 100 ATR FTIR Spectrometer (PerkinElmer, Waltham, MA, USA).

### 3.7. Encapsulation Efficiency (EE)

The EE was measured to quantify the proportion of curcumin captured by the NP system from what was introduced during synthesis; the colorimetric method reported by Pundarikakshudu et al. was employed [50]. Briefly, a standard curve for curcumin in ethanol (95%) was generated for a concentration range of 0 to 20 µM. The absorbance values for this concentration range were measured at 425 nm using a SpectraMax i3x plate reader (Molecular Devices, San Jose, CA, USA). These absorbance values were plotted against their respective concentrations to establish the standard curve. For the EE determination, 1 mL of CurNP was centrifuged at 10,000 rpm (Eppendorf 5810R [Fisher Scientific, Waltham, MA, USA]) for 15 min. The supernatant was then diluted in ethanol and its absorbance measured. The EE was calculated as follows:

$$EE\%=100\%-\left(\frac{Free\;Cur}{Initial\;Cur\;}\cdot 100\%\right)$$

*Free Cur* is the concentration of curcumin present in the supernatant while *Initial Cur* is the concentration of total curcumin added during the synthesis. The EE experiment was conducted in triplicates to obtain the average.

### 3.8. Fluorescence Spectroscopy

To assess the localization of curcumin within the CurNP system, the fluorescence spectra of free and encapsulated curcumin were recorded using a Jobin Yvon Nanolog Spectrofluorometer (Horiba Scientific, Piscataway, NJ, USA). The spectra were recorded with a Xenon lamp at 425 nm excitation/5 nm slit and the emission was collected from 450 to 700 nm. Before recording the spectra, the samples were washed and diluted according to the same protocol as noted for the SEM preparations.

### 3.9. Curcumin Degradation Rate

To evaluate the degradation rate of CurNPs and curcumin, their peak absorbances were measured at 425 nm every 60 min for 24 h. A Cary 300 Bio UV–Vis spectrophotometer with 1 cm quartz cuvette was employed for this method. Before the experiment, samples were diluted in DI water to a concentration of 40 µM of curcumin. All measurements for a given dispersion were measured using the same sample, contained within a cuvette that was left within the spectrophotometer for the entire duration of the experiment. The spectrophotometer was programmed to automatically obtain absorbance measurements at the indicated timepoints. The experiments were conducted in triplicates and statistical analyses were performed using simple linear regression in GraphPad Prism 9 (GraphPad Software Inc., Boston, MA, USA).

### 3.10. Curcumin Release Rate

The release of curcumin from NPs was evaluated over a period of 30 h. The samples were diluted in 50 mL of DI water to a concentration of 10 µM. Then, 5 mL aliquots were then taken at each time point and centrifuged at 12,000 rpm (Eppendorf 5414D) for 30 min. From this, 1 mL of the supernatant solution was transferred into a cuvette and its absorbance intensity measured at 425 nm using a Cary 300 Bio UV–Vis spectrophotometer. The resulting curcumin concentration was calculated using the same standard curve as described in the EE study. Finally, the drug release (%) at each time point was calculated using the following equation:

$$\%release\left(t_{min}\right)=\left(\frac{curcumin\;in\;supernatant}{initial\;curcumin\;in\;dispersion}\right)\times 100\%+\%initially\;released\;curcumin\;degraded\;by\;t_{min}$$

This equation accounts for the initially released curcumin that inevitably degrades by the time of measurement. This can be calculated by using the line equation of the slope for the degradation of free curcumin. It should be noted that this is a simplified model as it does not account for the degradation of subsequently released curcumin, only the curcumin released at the initial time point. Furthermore, the data sets were normalized to reflect ‘zero release’ at time zero. This normalization allows elimination of background signal from unencapsulated curcumin (~12% by subtraction of EE of 88% from 100%) and CurNPs too small to be pulled down during centrifugation.

### 3.11. Antioxidant Capacity with 2,2-Diphenyl-1-picryhyldrazyl (DPPH) Radical Scavenging Activity Assay

The antioxidant properties of CurNPs and its controls were elucidated from their DPPH scavenging activity. Reported methods with some modifications were followed [51,52]. Firstly, 280 µL of a prepared 80 µg/mL DPPH solution was added to wells of a 96-well plate. Then, 10 µL of absolute ethanol and 10 µL of the treatments were added to create a final concentration of 5 µM. Ascorbic acid was also included as a positive control due to its well-known antioxidant properties. Wells containing DPPH without treatments was also included to provide a negative control for calculations. Lastly, samples were incubated in dark conditions for an hour and the absorbance intensity was measured at 517 nm. The DPPH radical scavenging activity was calculated using the equation below:

$$\%DPPH\;scavenging\;activity\;=\;\frac{\left(DPPH\;abs\;-\;(treatment\;abs\;-\;treatment\;blank\;abs)\right)}{DPPH\;abs}\times 100\%$$

*Treatment abs* is the absorbance of wells containing DPPH and the treatments while *DPPH abs* is the absorbance of wells containing DPPH but without the treatments. The experiment was performed in triplicate and the statistical analysis was completed using one-way ANOVA in GraphPad Prism 9 (GraphPad Software Inc., Boston, MA, USA).

### 3.12. Cell Viability Assay

The safety profile of CurNPs was assessed by quantifying the viability of cells after exposure to treatments. An AlamarBlue (Thermo Scientific, Waltham, MA, USA) assay was performed following the guidelines from ISO 10993-5:2009 [47]. Cell types involved in PNI and subsequent PNR were chosen for this study: S16 rat Schwann cells (RSC-S16, ATCC CRL-2941, Manassas, VA, USA), murine macrophages (J774A.1, ATCC TIB-67, Manassas, VA, USA), and neuronal cells (SH-SY5Y, ATCC CRL-2266, Manassas, VA, USA). SH-SY5Y cells were cultured in DMEM/F12, whereas the J744A.1 and S16 RSC cells were cultured in DMEM. Both media were supplemented with 10% FBS and 1% streptomycin/penicillin. First, cells were cultured until 70 to 80% confluency in a T-75 flask and then seeded in a 96-well plate at a density of 10^4^ cells per well. The plates were incubated overnight in a humidified atmosphere at 37 °C and 5% CO_2_. Then, fresh medium with the treatments at a concentration range of 0 to 80 µM (curcumin) was added to wells, and the plates were incubated for another 24 h. In the case of TA–PVP, the concentration range was equivalent to the corresponding TA and PVP concentrations in the CurNPs. The treatments were then removed and fresh media containing 10% Alamar Blue (resazurin, 0.15 mg/mL) was added into all the wells. The plates were incubated for 2 h, and the fluorescence intensity was measured using a Tecan plate reader (Tecan Life Sciences, Zurich, Switzerland) with an Ex/Em of 560/590 nm. The cell viability was calculated using the following equation. The experiment was performed three times with biological triplicates for all conditions, resulting in an *n* = 9.

$$\%cell\;viability\;=\;\frac{fluorescence\;of\;treatment\;wells}{fluorescence\;of\;untreated\;wells\;(cells\;only)}\times 100\%$$

### 3.13. Confocal Fluorescence Microscopy for In Vitro Cellular Delivery of Curcumin

Confocal fluorescence microscopy was performed to determine the relative amount of curcumin in cells when delivered in the encapsulated versus free form. First, glass coverslips were plasma-treated and placed into a 6-well plate. SH-SY5Y (1 × 10^6^ cells/well), J774A.1 and RSC-S16 (both at 5 × 10^5^ cells/well) were then seeded onto the coverslips in individual plates and incubated overnight at 37 °C and 5% CO_2_. Then, the medium was replaced with media containing treatments at a curcumin concentration of 10 µM for J774A.1 cells, and 5 µM for RSC and SH-SY5Y cells. Following incubation for 24 h, cells were gently washed with PBS and then fixed with 4% paraformaldehyde solution for 10 min at room temperature. All paraformaldehyde residue was then removed by washing the cells with PBS three times. Following that, 1 mL of 10 µg/mL Hoechst solution was added into each well and allowed to incubate for 10 min at room temperature. Finally, cells were washed with PBS and mounted onto glass slides. The mounting medium used was 90% glycerol supplemented with 2 mg/mL L-ascorbic acid. The confocal images were generated from a z-stack of images acquired using a Keyence BZ-X800 fluorescence microscope (KEYENCE, Osaka, Japan) whereby the same exposure was applied to all samples and conditions. Curcumin and Hoechst were visualized in the green and blue channels, respectively.

The green fluorescence intensity in the acquired images was then quantified using the open-source Fiji software (version 2.9.0 for Java 8). For each experimental condition, the number of cells and global intensity were quantified, allowing for the calculation of mean fluorescence intensity per cell according to the equation shown below. The calculated mean fluorescence intensity of the ‘free curcumin’ and ‘untreated control’ groups were normalized against CurNPs given that it had the highest intensity. The experiments were conducted three times in triplicates resulting in an *n* = 9. Statistical analyses were performed using a one-way ANOVA in GraphPad Prism 9 (GraphPad Software Inc., Boston, MA, USA).

$$Mean\;fluorescence\;intensity\;=\;\frac{global\;intensity}{number\;of\;cells}$$

### 3.14. Hydrogen Peroxide (H_2_O_2_)_-_Induced Oxidative Stress and Viability Assay

The potential for CurNPs to rescue cells from oxidative stress induced by H_2_O_2_ exposure was assessed by conducting a cell viability assay, similar to a protocol reported by Mahakunakorn et al. [23]. Briefly, J744A.1 murine macrophages were seeded in a 96-well plate and incubated overnight at 37 °C and 5% CO_2_. Cells were treated with fresh media containing treatments for 6 h, and then exposed to fresh media containing H_2_O_2_ at a concentration of 100 µM for another 18 h. After incubation, fresh media with 10% Alamar Blue (0.15 mg/mL) was added to all the wells and incubated for 2 h. Finally, fluorescence intensity was measured at an Ex/Em of 560/590 nm to determine cell viability using the equation described in the cell viability method section. The experiments were conducted in triplicates and statistical analyses were performed using a one-way ANOVA in GraphPad Prism 9 (GraphPad Software Inc., Boston, MA, USA).

## 4. Conclusions

The overarching goal of these studies was to create a NP platform to encapsulate curcumin and improve its physicochemical properties, ultimately optimizing delivery to cells and improving therapeutic efficacy. Overall, the results demonstrate the successful synthesis of CurNPs with a high encapsulation efficiency and improved colloidal stability of curcumin in aqueous solutions. Further, it was shown that the encapsulation of curcumin by TA–PVP significantly decreased its degradation rate by 20% and allowed sustained release over a period of at least 20 h. It is anticipated that the observed trend of release kinetics would be retained over longer periods as the NP carrier degrades. Future studies should be performed for periods relevant to in vivo studies of PNI and NC applications.

These improved physical and chemical characteristics offered by the NP system successfully translated into increased delivery of curcumin into cell models for PNI and PNR, as demonstrated by the confocal fluorescence microscopy studies of J77A.1, SH-SY5Y, and RSC-S16 cells. It can be inferred that the NP’s ability to protect curcumin from degradation and provide sustained release allows for a higher amount to remain present in cells after 24 h. Additionally, the cell viability studies shown here not only provide evidence of the CurNP’s biocompatibility but also its ability to rescue J774A.1 macrophages from excessive oxidative stress induced by hydrogen peroxide. This protection against ROS would be an important attribute to consider in the incorporation of bioactive agents for NC applications. Future studies should be conducted to assess CurNPs embedded into NCs and the potential to treat PNI in animal models but these results nonetheless provide evidence to support improved drug delivery to cells.

## 5. Patents

Based on this work the authors have submitted patent application US20230295436A1. There are no other conflicts to declare.

## Figures and Tables

**Table 1:** Optimization of the EE by increasing molar ratios TA and PVP to curcumin and reducing the overall volumes.

Molar Ratio (Cur:TA:PVP)	Water (mL)	Ethanol, 95% (mL)	Hydrodynamic Size Average (nm)	Encapsulation Efficiency (%)
1:2:0.021	14	1	207	66
1:2:0.021	9	1	210	77
1:3:0.032	14	1	229	66
1:3:0.032	9	1	214	82
1:3.3:0.035	9	1	220	88

## Data Availability

The data presented in this study are available on request from the corresponding author.

## Supplementary Materials

The following supporting information can be downloaded at: https://www.mdpi.com/article/10.3390/molecules29102281/s1, References [53,54,55,56,57] are cited in Supplementary Materials.
