# Supplementary material for: Design and Development of a Polymeric-Based Curcumin Nanoparticle for Drug Delivery Enhancement and Potential Incorporation into Nerve Conduits
Source: Molecules. 2024 May 12;29(10):2281. doi: 10.3390/molecules29102281 (PMC11124517; doi:10.3390/molecules29102281)
Supplement: Supplementary file 1 [file molecules-29-02281-s001.zip › molecules-2966824-supplementary.pdf]

Design and Development of a Polymeric-based Curcumin Nanoparticle for Drug Delivery enhancement and Potential Incorporation into Nerve Conduits

Giuliana Gan Giannelli,[b,c] Edwin Davidson,[a,b,] Jorge Pereira,[a,b,] and Swadeshmukul Santra[a,b,c,*]

[a]*Department of Chemistry, University of Central Florida, Orlando, FL, 32826, USA.*
[b]*NanoScience Technology Center, University of Central Florida, Orlando, FL, 32826, USA.*
[c]*Burnett School of Biomedical Sciences, University of Central Florida, Orlando, FL, 32826, USA.*

[*]*Corresponding authors e-mails swadeshmukul.santra@ucf.edu,*

Table of Contents

Table S1. Optimization of the encapsulation efficiency (EE%) with different molar ratios of TA and PVP to curcumin. A plateau in EE% was achieved at molar ratio 1: 2: 0.021 (Cur: TA: PVP). 2
Table S2. FTIR spectra of Cur, CurNP, and TA-PVP. 3

Figure S1. Image of curcumin dispersed in water in its free versus encapsulated form. Free curcumin exhibits significant precipitation in water while CurNPs have notably increased colloidal stability. 4
Figure S2. (A) SEM image of unloaded TA-PVP nanoparticles (B) DLS histogram showing the hydrodynamic diameter size distribution of unloaded TA-PVP nanoparticles. (C) Zeta potential charge distribution of unloaded nanoparticles in aqueous solution. 5
Figure S3. DPPH radical scavenging activity of treatments. Treatments were prepared to a final concentration corresponding to 5 µM of curcumin, and 5 µM of ascorbic acid (positive control). Data are represented as the mean ± standard deviation (n=3). Statistical analysis was performed using one-way ANOVA (****$p<0.0001$). 6
Figure S4. H_2O_2-induced oxidative stress and cell rescue by treatments on J774A.1 murine macrophages. Cells were pre-treated with CurNPs, TA-PVP, or free curcumin for 6 hours, and then treated with fresh media containing 100 µM H_2O_2 for 18 hours. Cells were then treated with 10% resazurin for 2 hours, and fluorescence was read at an ex/em of 560/ 590 nm. There appears to be some protection against oxidative stress when cells are treated with 10 µM of CurNPs. Data are represented as mean ± standard deviation (n=3). A one-way ANOVA was performed to determine any statistically significant differences compared to the H_2O_2 control (**$p<0.01$). 7

Table S1. Optimization of the encapsulation efficiency (EE%) with different molar ratios of TA and PVP to curcumin. A plateau in EE% was achieved at molar ratio 1: 2: 0.021 (Cur: TA: PVP).

Molar ratio of formulation (Cur: TA:PVP)	Water (mL)	Ethanol 95% (mL)	Hydrodynamic Size Average (nm)	Zeta Potential (mV)	Polydispersity Index (PDI)	Encapsulation Efficiency (%)
1: 1: 0.01	14	1	150	-23	0.5	36
1: 1.3: 0.014	14	1	148	-35	0.4	35
1: 1.7: 0.018	14	1	169	-38	0.4	52
1: 2: 0.021	14	1	207	-33	0.5	66
1: 2.3: 0.025	14	1	230	-40	0.5	68
1: 2.7: 0.028	14	1	208	-38	0.5	68
1: 3: 0.032	14	1	229	-26	0.6	66

Table S2. FTIR spectra of Cur, CurNP, and TA-PVP.

**Literature characteristic absorption band range** [1-5]	**Assignment of absorption bands**	**Cur (cm^{-1}) [3,4}**	**CurNP (cm^{-1})**	**TA-PVP (cm^{-1}) [1,2,5]**
3550 – 3200	O-H stretch	3508	3650	-
1730 – 1705	C=O stretch	-	1718	1718
1670 – 1600	C=C stretch	1628	1628	1655
1350 – 1480	benzene ring stretches	1597	1597	-
1465 – 1380	C-H bend	1428	1428	-
1452 – 1420	Aromatic C-C bend	-	1450	1450
1310 – 1250	Aromatic C-O stretch	1278	1278	-
1300 – 1100	C-O stretch	-	1197	1197
1050 – 1020	C-O-C stretch	1024	1030	1030
800 – 646	C=C benzene ring distortion	-	760	760
570 – 650	N-C=O bend	-	647	647

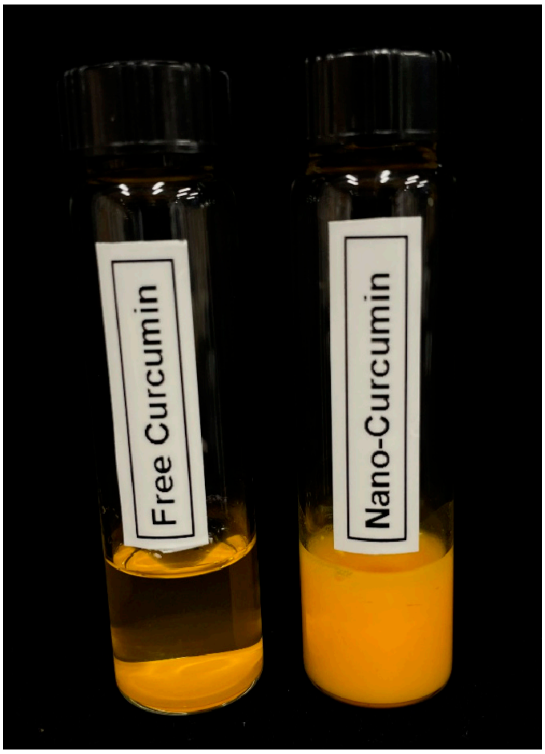

Figure S1. Image of curcumin dispersed in water in its free versus encapsulated form. Free curcumin exhibits significant precipitation in water while CurNPs have notably increased colloidal stability.

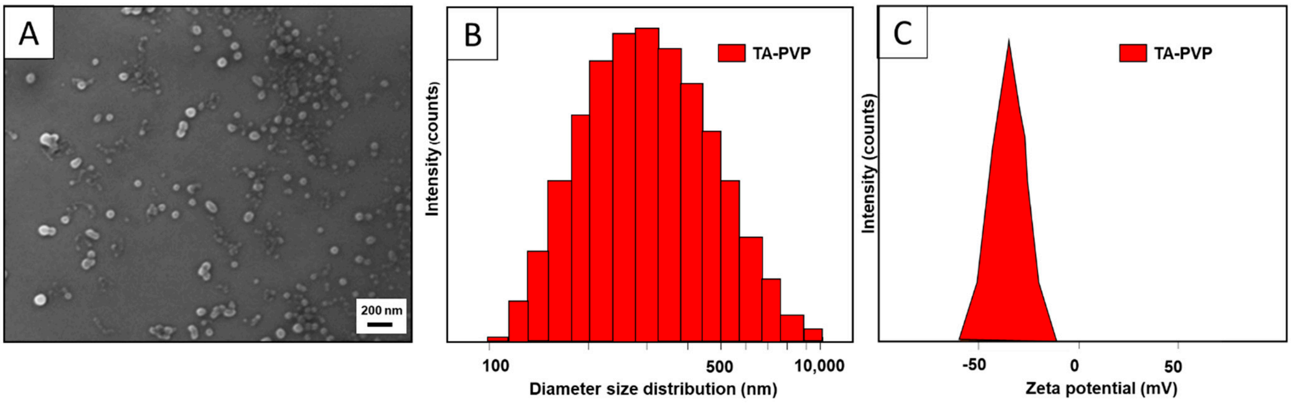

Figure S2. (A) SEM image of unloaded TA-PVP nanoparticles (B) DLS histogram showing the hydrodynamic diameter size distribution of unloaded TA-PVP nanoparticles. (C) Zeta potential charge distribution of unloaded nanoparticles in aqueous solution.

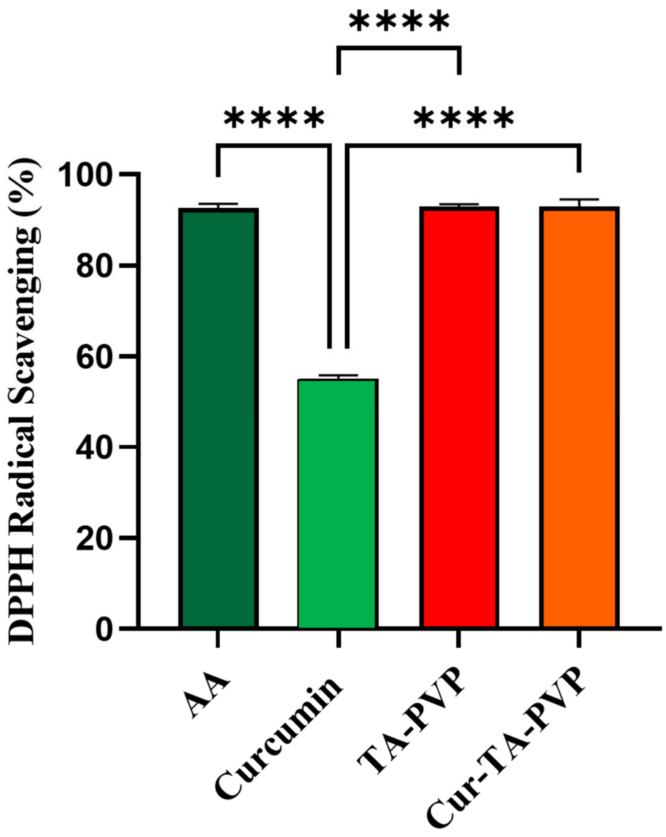

Figure S3. DPPH radical scavenging activity of treatments. Treatments were prepared to a final concentration corresponding to 5 µM of curcumin, and 5 µM of ascorbic acid (positive control). Data are represented as the mean ± standard deviation (n=3). Statistical analysis was performed using one-way ANOVA (****p<0.0001).

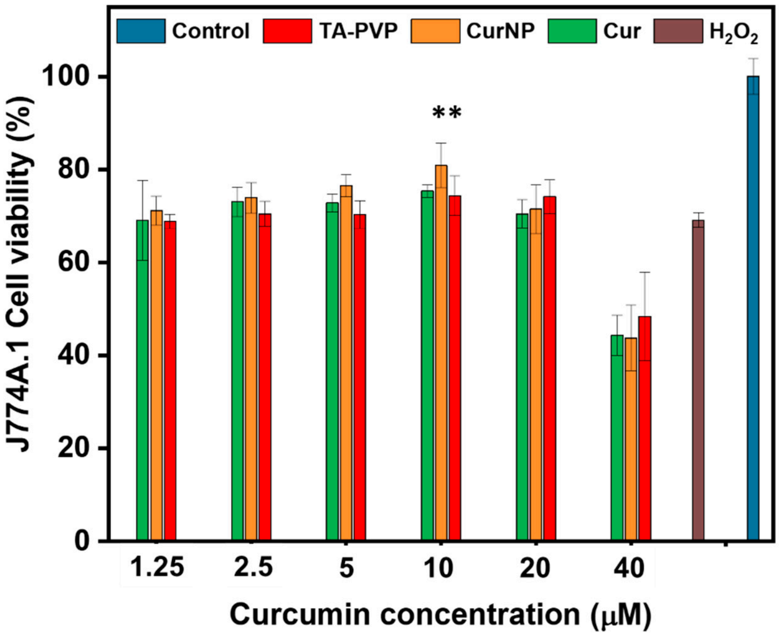

Figure S4. H_2O_2-induced oxidative stress and cell rescue by treatments on J774A.1 murine macrophages. Cells were pre-treated with CurNPs, TA-PVP, or free curcumin for 6 hours, and then treated with fresh media containing 100 μM H_2O_2 for 18 hours. Cells were then treated with 10% resazurin for 2 hours, and fluorescence was read at an ex/em of 560/ 590 nm. There appears to be some protection against oxidative stress when cells are treated with 10 μM of CurNPs. Data are represented as mean ± standard deviation (n=3). A one-way ANOVA was performed to determine any statistically significant differences compared to the H_2O_2 control (**$p<0.01$).

References

1. Socrates, G., *Infrared and Raman characteristic group frequencies: tables and charts*. 2004: John Wiley & Sons.
2. Pantoja-Castro, M.A. and H. González-Rodríguez, *Study by infrared spectroscopy and thermogravimetric analysis of tannins and tannic acid.* Revista latinoamericana de química, 2011. **39**(3): p. 107-112.
3. Yallapu, M.M., M. Jaggi, and S.C. Chauhan, *β-Cyclodextrin-curcumin self-assembly enhances curcumin delivery in prostate cancer cells.* Colloids and surfaces B: Biointerfaces, 2010. **79**(1): p. 113-125.
4. Chen, X., et al., *The Stability, Sustained Release and Cellular Antioxidant Activity of Curcumin Nanoliposomes.* Molecules, 2015. **20**(8): p. 14293-14311.
5. Basha, M.A.-F., *Magnetic and optical studies on polyvinylpyrrolidone thin films doped with rare earth metal salts.* Polymer journal, 2010. **42**(9): p. 728-734.
